# Evaluation of the validated intraoperative bleeding scale in liver surgery: study protocol for a multicenter prospective study

**DOI:** 10.3389/fsurg.2023.1223225

**Published:** 2023-10-02

**Authors:** Daniel Aparicio-López, José Manuel Asencio-Pascual, Gerardo Blanco-Fernández, Esteban Cugat-Andorrá, Miguel Ángel Gómez-Bravo, Santiago López-Ben, Elena Martín-Pérez, Luis Sabater, José Manuel Ramia, Mario Serradilla-Martín

**Affiliations:** ^1^ Department of Surgery, Hospital Universitario Miguel Servet, Zaragoza, Spain; ^2^ Department of Surgery, Hospital Universitario Gregorio Marañón, Madrid, Spain; ^3^ Department of Surgery, Hospital Universitario de Badajoz, Badajoz, Spain; ^4^ Department of Surgery, Hospital Universitario Mutua de Terrassa, Terrassa, Spain; ^5^ Department of Surgery, Hospital Universitario German Trials I Pujol, Barcelona, Spain; ^6^ Department of Surgery, Hospital Universitario Virgen del Rocío, Seville, Spain; ^7^ Department of Surgery, Hospital Universitario Dr. Josep Trueta, Girona, Spain; ^8^ Department of Surgery, Hospital Universitario La Princesa, Madrid, Spain; ^9^ Department of Surgery, Hospital Clínico Universitario, INCLIVA, Valencia, Spain; ^10^ Department of Surgery, Hospital General Universitario Dr. Balmis, ISABIAL, Universidad Miguel Hernández, Alicante, Spain; ^11^ Department of Surgery, Instituto de Investigación Sanitaria Aragón, Hospital Universitario Miguel Servet, Zaragoza, Spain

**Keywords:** liver surgery, predictive score, intraoperative bleeding, surgical hemostasis, hemostatic agent

## Abstract

**Background:**

Surgical hemostasis has become one of the key principles in the advancement of surgery. Hemostatic agents are commonly administered in many surgical specialties, although the lack of consensus on the definition of intraoperative bleeding or of a standardized system for its classification means that often the most suitable agent is not selected. The recommendations of international organizations highlight the need for a bleeding severity scale, validated in clinical studies, that would allow the selection of the best hemostatic agent in each case. The primary objective of this study is to evaluate the VIBe scale (Validated Intraoperative Bleeding Scale) in humans. Secondary objectives are to evaluate the scale's usefulness in liver surgery; to determine the relationship between the extent of bleeding and the hemostatic agent used; and to assess the relationship between the grade of bleeding and postoperative complications.

**Methods:**

Prospective multicenter observational study including 259 liver resections that meet the inclusion criteria: patients scheduled for liver surgery at one of 10 medium-high volume Spanish HPB centers using an open or minimally invasive approach (robotic/laparoscopic/hybrid), regardless of diagnosis, ASA score <4, age ≥18, and who provide signed informed consent during the study period (September 2023 until the required sample size has been recruited). The participating researchers will be responsible for collecting the data and for reporting them to the study coordinators.

**Discussion:**

This study will allow us to evaluate the VIBe scale for intraoperative bleeding in humans, with a view to its subsequent incorporation in daily clinical practice.

**Clinical Trial Registration:**

https://clinicaltrials.gov/ct2/show/NCT05369988?term = serradilla&draw = 2&rank = 3, [NCT0536998].

## Introduction

Surgical hemostasis has become one of the key principles for the advancement of surgery (1). The use of hemostatic agents is standard in many surgical specialties (2–6), especially in major surgeries (7, 8). However, inadequate hemostasis significantly increases the risk of perioperative morbidity and mortality (9, 10), healthcare costs, and the use of resources (11–14). The need for hemostasis has led to the development of a range of topical hemostatic agents (15–17) to supplement hemostasis efforts.

Generally, bleeding from solid organs can be divided into two types: (a) First, bleeding which is due to inadequate control of surgical field and which requires either suture control or resection of the organ to stop the bleeding. The available hemostatic agents are essentially always ineffective with this degree of bleeding; (b) The second type of bleeding may be described as “medical”, which is due to coagulopathy or that which is controlled by packing.

Clinical studies that have analyzed the use of topical hemostats have not used standardized definitions or classifications to determine the severity of intraoperative bleeding (18–23), partly due to a lack of consensus on its definition. This has meant that many surgeons do not select the best hemostats for specific bleeding situations, and the results are unsatisfactory (8). Using standardized criteria, the results of clinical studies can be compared in order to determine the effectiveness of different hemostatic agents.

The Food and Drug Administration (FDA) (24) recommends the use of a bleeding severity scale validated in clinical studies of hemostatic agents. The VIBe scale (Validated Intraoperative Bleeding Scale) for intraoperative bleeding was developed in 2017 (25) and is approved by the FDA as a clinical report scale (Table 1). It has been validated by a large number of expert surgeons from all specialties, but only in animal models; it has not yet been clinically validated in humans. Depending on the degree of intraoperative bleeding, the scale recommends a specific type of hemostatic.

**Table 1:** Validated bleeding severity scale (25).

Grade	Visual presentation	Anatomic appearance	Qualitative description	Visually estimated rate of blood loss (ml/min)
0	No bleeding	No bleeding	No bleeding	≤1.0
1	Ooze or intermittent flow	Capillary-like bleeding	Mild	>1.0–5.0
2	Continuous flow	Venule and arteriolarlike bleeding	Modarate	>5.0–10.0
3	Controllable spurting and/or overwhelming flow	Noncentral venous- and arterial-like bleeding	Severe	>10.0–50.0
4	Unidentified or inaccessible spurting or gush	Central arterial- or venous-like bleeding	Life threatening	>50.0

Liver surgery is one of the surgeries in which intraoperative bleeding is most important. In Spain, around 5,000 liver resections are performed annually, not including liver transplants. It is, therefore, a perfect setting in which to evaluate the applicability of the VIBe scale of intraoperative bleeding severity in humans.

To assess the feasibility of a prospective clinical study in liver surgery, the applicability of the VIBe scale was evaluated by 47 liver surgeons from 10 medium-high volume centers who viewed the 14 original video recordings used by Lewis et al. (25) for the validation of the original scale. The scale achieved a mean intraobserver agreement of 0.985 and an interobserver agreement of 0.929; neither score was influenced by surgeon experience or volume of surgeries per year.

With these preliminary results, based on the video classification exercise, we conclude that the VIBe scale is a useful instrument for liver surgeons assessing severity of bleeding. Therefore, it can be used to assess the severity of intraoperative bleeding in actual liver surgery.

This prospective multicenter study carried out at centers in Spain that perform liver surgery aims to evaluate a scale that allows the grading of intraoperative bleeding in surgery, and to determine whether there is a relationship between the maximum bleeding grade and complications.

## Methods

### Hypothesis and primary/secondary objectives

To date, the VIBe scale for the severity of intraoperative bleeding has only been validated in an animal model (25). Intraoperative bleeding occurs frequently in liver surgery and can have a major impact on the postoperative results. Liver surgery is thus an ideal setting for applying the VIBe scale in humans.

The primary objective is to evaluate the VIBe scale for intraoperative bleeding in liver surgery. The secondary objectives are to assess the usefulness of the scale in this type of surgery; to determine the relationship between bleeding grade and hemostatic method used; and to determine the relationship between the maximum bleeding grade reached on the VIBe scale and postoperative complications.

### Variables analyzed

The key point of the study is to determine the grade of intraoperative bleeding evaluated with the VIBe scale. In the initial study by Lewis et al. (25), the only variable analyzed was the volume of intraoperative bleeding, in order to establish the grade of bleeding. In our study, we intend to evaluate the usefulness of this scale in daily clinical practice. For this reason, regardless of the volume of bleeding, we collect all those variables that may be related to intraoperative bleeding.

Thus, we will evaluate the VIBe bleeding scale by examining how well its recommended hemostatic interventions align with the actual usage of hemostatics in typical clinical scenarios. This evaluation will involve a calibration assessment using the Hosmer-Lemeshow test, which examines the agreement between predicted probabilities of hemostatic usage based on the scale and observed clinical outcomes. Additionally, we will utilize calibration plots to visually depict the consistency between predicted and observed outcomes. This analysis will provide valuable insights into the scale's accuracy and alignment with real-world practice, helping to determine its practical effectiveness in guiding hemostatic interventions (26).

Because there may be numerous bleeding episodes during a hepatectomy, it is impossible to measure each one individually. The collaborating researchers propose its assessment at two selected time points: the moment of greatest bleeding, and the end of the surgery. An intraoperative record sheet is prepared and completed according to the comments of the main or assistant surgeon and will be reviewed and finally signed by the main surgeon at the end of the procedure. Similarly, blood loss (the amount of fluid in the suction container minus the volume of liquid used for flushing), the units of blood transfused and the lowest hemoglobin value during admission (compared to the baseline rate) will be quantified. The rest of the data will be collected at the time of discharge and 90 days after surgery.

Patient comorbidities will be classified using the Charlson Comorbidity Index (27). Likewise, the patient's coagulation status (PT/PTT/INR/TEG) and treatments that may alter it (anticoagulants and antiaggregants) will be collected. Postoperative complications will be classified using the Clavien-Dindo Classification of Surgical Complications (28) and the Comprehensive Complication Index (CCI) (29). Major complications are those defined as Clavien-Dindo grade IIIa or higher. To record complications, the medical and nursing notes from each electronic or physical record of each patient included in the project will be compared. Intraoperative complications will be measured according to the Satava classification (30). For the specific complications of liver surgery, the definitions of the International Study Group of Liver Surgery (ISGLS) for liver failure (31) and postoperative bleeding (32) will be used. Complications, readmissions and mortality will be measured at postoperative day 90, through the medical history and, if necessary, by telephone communication with the patient or relatives (Supplementary Files S1, S2).

We plan to examine the relationship between the maximum bleeding grade and the occurrence of complications through logistic models, calculating odds ratios to quantify the risk of experiencing complications in relation to different bleeding grades, particularly for dichotomous variables. For variables that evolve over time, we will employ Cox proportional hazard models to estimate hazard ratios, allowing us to understand the impact of bleeding grades on the timing of complications.

### Study design

This study is a prospective observational multicenter study lasting 12 months (the period can be lengthened if the stipulated number of patients has not been reached) involving 10 Spanish HPB Surgery units with medium-high volume of liver surgery (Supplementary File S3). Medium-volume centers will be considered those that perform 40–60 liver surgeries per year, and high-volume centers those with more than 60 per year (33). Surgeons from participating centers will receive training prior to the start of the study on how to properly categorize the degree of intraoperative bleeding from the videos in the Lewis article (25).

Patients operated on either via open surgery or via a minimally invasive approach will be included; thus, the VIBe scale will also be evaluated for minimally invasive surgery. In this sense, an analysis by subgroups of “open surgery” vs. “minimally invasive surgery” will be performed.

### Inclusion and exclusion criteria and rationale for the study population

Participants meeting all the following inclusion criteria are eligible for the study:

- Patients scheduled for liver surgery
- Open or minimally invasive approach (laparoscopic/robotic/hybrid)
- Any diagnosis
- ASA score <4
- Age ≥ 18
- Provision of signed informed consent

Patients with any of the following exclusion criteria will not take part:

- Contraindications for liver surgery
- Emergency surgical interventions
- Age <18 years
- Failure to provide signed informed consent
- Loss to follow-up and impossibility of completing the data record.

### Recruitment, screening and informed consent procedure

This registry includes all the patients undergoing liver surgery at the participating Spanish centers in the study period who meet the inclusion criteria. All ten are reference centers which participated in the first phase of the study with the validation based on the videos. The registry will remain open for a further three months after the required sample size has been recruited to record postoperative morbidity and mortality at 90 days.

When confirming their participation, the participating centers will appoint a contact person.

The study will be presented to the patients during the first preoperative consultation by the investigators at each participating hospital. The investigators will explain to each participant the nature of the study, its purpose, the procedures involved, the expected duration, the potential risks and benefits, and any discomfort it may entail. Participants will be informed that their participation in the study is voluntary and that they may withdraw at any time, and that withdrawal of consent will not affect their subsequent medical assistance and treatment. They will be notified that their medical records may be examined by authorized individuals other than their treating physician. They will be given a participant information sheet and a consent form describing the study and providing sufficient information for them to make an informed decision about whether to take part. Patients will have the opportunity to ask any questions they may have.

Formal consent will be obtained from participants using the approved consent form, before they undergo any of the study procedures. The consent form will be signed and dated by the investigator or designee at the same time as the participant signs. A copy of the signed informed consent will be given to the participant. The consent form will be retained as part of the study records. The informed consent process will be documented in the patient file and any discrepancies with regard to the process described in this protocol will be explained (Supplementary File S4).

### Planning

Data collection will begin in September 2023.

### Withdrawal and discontinuation

As it is a prospective registry that does not involve any intervention in the patient, consecutive cases that meet the inclusion criteria operated on at the participating centers will be included in the study. Only those patients who, having signed the informed consent, refuse to participate in the study prior to the intervention will be excluded; in this case they will be replaced by a new patient until the required sample size is obtained.

### Follow-up

In liver surgery, complications may occur beyond 30 postoperative days. Therefore, the international scientific community has agreed to record complications at 90 days.

### Statistical analysis and sample size calculation

Depending on the number of cases contributed by each collaborating hospital, a retrospective analysis will be carried out to detect the power of the differences observed in the data. The variables of interest will be displayed in univariate and bivariate tables according to study group. For comparisons between groups, parametric (t-student, ANOVA) and non-parametric (Mann-Whitney, Kruskal-Wallis) tests will be used in continuous variables depending on their distribution, and Fisher or chi-square tests for categorical variables.

To investigate the relationship between different variables, correlation analysis and/or linear regression and bi- or multivariate logistic analysis will be used. In addition, the longitudinal variation of certain variables of interest will be studied with Kaplan-Meier estimators and bi- or multivariate analysis using Cox models. The analyses will be performed with the R statistical software package. Statistical significance will be considered for values of *p* < 0.05 (34–36).

Based on an estimated population size of 5,000 liver surgeries per year in Spain, a sample size of 259 cases will be selected to achieve a margin of error of 5% with a confidence level of 90%. Based on the normal distribution, the sample size *n* and the margin of error *E* are given by:

$$\begin{array}{c} x\;=\;Z\;(c\;/\;100)2r(100\text{-}r)\;\\ n\;=\;N\;x/((N\text{-}1)E2+x)\;\\ E\;=\;\mathrm{Sqrt}\;[\left(N\;\text{-}\;n)x/n(N\text{-}1\right)]\; \end{array}$$

Where *N* is the size of the population, r is the fraction of responses (set at 50% as the most conservative assumption) and Z (c/100) is the critical value for the confidence level c (34–36).

### Data record

Each participating center will appoint a contact person responsible for data collection and for all communication with the study coordinators. Subsequently, each contact person will receive a login code and passwords for the online electronic case report form environment (REDCap®, University of Vanderbilt, Nashville, Tennessee, United States). Each data collector will receive a separate login account from which the lead study coordinators can monitor all activity. All data will be recorded in accordance with the Good Clinical Practice (GCP) guidelines.

The variables collected in this form are shown in Supplementary Files S1, S2.

### Authorship and publication policy

Authorship will be based on the guide of the “International Committee of Medical Journal Editors” (37). The study coordinators will occupy the first and last positions of authorship, followed by the principal investigators of each center. A collaborative group will be created that will include the rest of the participating surgeons from each center in the study. The data obtained will be communicated to the local research ethics committee in accordance with Spanish legislation and then published.

The data generated by the study will be sent to journals with a high impact factor indexed in the Journal Citation Reports (JCR). The results will also be presented as communications at international HPB surgery conferences (IHPBA/ E-AHPBA congress).

## Discussion

### Anticipated results

Despite the extensive literature published on surgery, there is no evidence at present to help create validated scales that quantify the severity of intraoperative bleeding and thus select the best hemostatic procedure for each case. Likewise, the Spanish Health System (where this study is to be carried out) does not impose limitations on the use of intraoperative surgical material (i.e., local hemostatics, types of suture, sealants, mechanical staplers, etc.). The choice of the method for each situation is left the discretion of the liver surgeon. All this has repercussions for patient safety; it may lead to the inappropriate use of healthcare resources and increased costs, and it may increase postoperative morbidity and mortality. Against this background, liver surgery appears to be an ideal setting for applying the VIBe scale for grading intraoperative bleeding in humans.

The authors expect to find an adequate clinical correlation of the VIBe scale with the clinical results of intraoperative bleeding in liver surgery at two time points: the moment of maximum bleeding and the end of the surgery. This will allow its evaluation and its later incorporation in daily clinical practice. In addition, intraoperative bleeding is expected to correlate with postoperative complications.

### Overall ethical considerations

As there are no previous references in the literature to the existence of a validated scale that categorizes intraoperative bleeding in liver surgery, the study design (a ospective multicenter registry lasting 12 months) should have good internal validity. Medium/high volume HBP surgery centers in Spain will participate in this multicenter study, thus obtaining a large sample size that will allow us to collect highly detailed, representative, and reliable information and will increase the generalizability of the results (external validity).

If the results are favorable, the VIBe scale will be considered validated as a severity scale for intraoperative bleeding in clinical studies that allows the selection of the best hemostatic method in each case, in compliance with the recommendations of international organizations. The results of the study can be implemented and promptly transferred into daily clinical practice.

The need for research in this field is clear, since the issue of intraoperative bleeding, more specifically in liver surgery, has not been resolved and there is no consensus among surgeons. This situation leads to a notable heterogeneity in clinical practice. The results of this study would go beyond their mere scientific interest, since they will have a direct impact on the outcomes of patients undergoing liver surgery. Given that the indications for liver resections are rising, the importance of the results will increase correlatively.

### Risk-benefit assessment

This is a prospective registry of patients undergoing liver surgery. Liver surgery is a common procedure that does not require the performance of any unusual measures.

## Limitations

The study has a multicenter design, and so there may be selection or information biases if centers apply different criteria and surgical indications. The quality of the outcome measurement is limited by the possible heterogeneity between centers in their assessment of intraoperative bleeding.

## Conclusion

It is hoped that this study will provide new insights into the impact of intraoperative bleeding in liver surgery. It is designed to validate a tool that allows the categorization of bleeding and the selection of the best individualized hemostatic method available for each patient.

## Ethics and dissemination

This study has the approval of the Research Ethics Committee of Aragon (CEICA). Session of 01/26/2022, Acta No. 02/2022. C.P.—I.C. PI21/510. All included patients will provide written consent to participate in this study.

The dissemination of the study will be carried out through the Division of HPB Surgery of the Spanish Association of Surgeons (AEC), and by the Spanish Chapter of the International Association of Hepato-Pancreato-Biliary Surgery (CE-IHPBA).

## Data Availability

The original data will be included in the articles and **Supplementary Materials**. Further requests for data may be addressed to the corresponding author.

## Supplementary material

Supplementary File S1 Study variables.

Supplementary File S2 Schedule of assessments.

Supplementary File S3 Participating centers.

Supplementary File S4 Patient information document and individual informed consent.
