# Supplementary material for: Evaluation of the validated intraoperative bleeding scale in liver surgery: study protocol for a multicenter prospective study
Source: Front Surg. 2023 Oct 2;10:1223225. doi: 10.3389/fsurg.2023.1223225 (PMC10577188; doi:10.3389/fsurg.2023.1223225)
Supplement: Supplementary File S1 — Study variables. [file Table1.docx]

**SUPPLEMENTARY FILES**

**SUPPLEMENTARY FILE S1. Study variables.**

***DEMOGRAPHIC DATA***

| Hospital |  |
| --- | --- |
| Location |  |
| Patient identification number |  |
| Date of birth | month/day/year |
| Sex | Male  Female |
| Weight | kg |
| Height | m |
| BMI | Kg/m^2^ |
| ASA score | I  II  III  IV |
| Charlson Comorbidity Index | Diabetes without target organ involvement  Diabetes with target organ involvement.  Myocardial infarction  Coronary heart disease  Peripheral vascular disease  Cerebrovascular disease  Arterial hypertension  Alcoholism  Thromboembolic disease  Arrhythmia  Dementia  COPD  Connective tissue disease  Peptic ulcer  Mild liver disease (without portal hypertension, includes chronic hepatitis)  Hemiplegia  Moderate-severe kidney disease  Leukemia (acute or chronic)  Lymphoma  Moderate or severe liver disease  Solid tumor with metastasis  AIDS (not just HIV positive) |
| Preoperative coagulation status | PT  PTT  INR  TEG |
| Does the patient have any coagulopathy? | No / Yes (please describe/ specify) |
| Preoperative medication | **Antiplatelet agents**  Acetylsalicylic acid  Carbasalate  Citazole  Clopidogrel  Dipyridamole  Sulfinpyrazone  Ticlopidine  Triflusal  **Oral anticoagulants**  Acenocoumarol  Apixaban  Dabigatran  Edoxaban  Phenprocoumon  Warfarin  Rivaroxaban  **Heparin and derivatives**  Bemiparin  Dalteparin  Enoxaparin  Nadroparin  Tinzaparin |
| Surgical history | Cholecystectomy  Hepatectomy  Other supramesocolic surgery  Inframesocolic surgery  Others  No |
| Underlying disease | Liver metastasis from colorectal cancer  Liver metastasis from neuroendocrine tumor  Liver metastasis from non-colorectal non-neuroendocrine tumor  Hepatocarcinoma  Cholangiocarcinoma  Adenoma  Focal nodular hyperplasia  Others |
| Time elapsed from diagnosis to start of treatment (days) | Days |
| Neoadjuvant chemotherapy | Yes  No |
| Type of chemotherapy | Describe chemotheraphy regimen |

***INTRAOPERATIVE DATA***

| Date of surgery | month/day/year |
| --- | --- |
| Previous portal embolization | Yes  No |
| Previous volumetry | Yes  No |
| Functional residual volume | cc |
| Two-stage surgery | Yes  No |
| Reverse surgery | Yes  No |
| Surgical approach | Open (laparotomy)  Laparoscopy  Robotic  Conversion |
| Type of liver (macroscopic) | Normal  Steatosis  Fibrosis  Chemotherapy-induced liver injury Cirrhosis |
| Liver segments affected (intraoperatively) | I  II  III  IVa  IVb  V  VI  VII  VIII |
| Type of surgery | Left lateral sectionectomy (2 and 3)  Bisegmentectomy (5 and 6)  Right anterior sectionectomy (5 and 8)  Right posterior sectionectomy (6 and 7)  Left hepatectomy (2, 3 and 4)  Right hepatectomy (5, 6, 7 and 8)  Central hepatectomy (4, 5 and 8)  Extended right hepatectomy (4, 5, 6, 7 and 8)  Extended left hepatectomy (2, 3, 4, 5 and 8)  Limited resection 1  Limited resection 2  Limited resection 3  Limited resection 4a  Limited resection 4b  Limited resection 5  Limited resection 6  Limited resection 7  Limited resection 8  Anatomical resection segment 1  Anatomical resection segment 2  Anatomical resection segment 3  Anatomical resection segment 4a  Anatomical resection segment 4b  Anatomical resection segment 5  Anatomical resection segment 6  Anatomical resection segment 7  Anatomical resection segment 8 |
| Other associated procedures | Radiofrequency  Microwave  Atypical resection  Others |
| Operative time (from skin to skin) | min |
| Bleeding | mL |
| Units of blood transfused |  |
| Units of platelets transfused |  |
| Units of fresh frozen plasma transfused |  |
| Extent of bleeding (major intraoperative bleeding) | 0: no bleeding (<1 mL / min)  1: mild (1-5 mL / min)  2: moderate (5-10 mL / min)  3: severe (10-50 mL / min)  4: very severe (> 50 mL / min) |
| Type of hemostasis performed | Manual compression  Clips  Electrocautery  Aquamantys  Ligasure/Harmonic/Thunderbeat/Other device  Suture  Application of hemostatic agent |
| Hemostatic agents used | **PASIVES**  ***Oxidized cellulose***  Surgicel (Original, Fibrilar, Nuknit, Snow)  Gelitacel  Pahacel  Traumastem  Oxitamp  Kanstat  Reoxcel  Curacel  Equitamp / Equicel  Cellistypt  ***Porcine gelatin***  Gelfoam  Espongostan  Gelita / Gelitaspon  Surgispon  Curaspon  Cutanplast  Hemospon  Surgiflo without thrombin  ***Bovine collagen***  Avitene  Helitene  Hemacoll  Hemotese  Instat  Lyostypt  Novacol  Sangustop  ***Equine collagen***  Collatamp  Genta-Coll  GentaFleece  ***Polysaccharide powders***  PerClot  Arista  4DryField  Haemocer  ***Oxidized cellulose powders***  Surgicel poder  ***Synthetic peptides***  Purastat  **ACTIVES**  ***Fibrin glue***  Tisseel  Veraseal  Vivostat  ***Thrombin and gelatin matrix***  Floseal  Surgiflo with thrombin  ***Advanced patches***  Tachosil  Hemopatch  Veriset |
| Clamping time | min |
| Extent of bleeding (at the end of surgery) | 0: no bleeding (<1 mL / min)  1: mild (1-5 mL / min)  2: moderate (5-10 mL / min)  3: severe (10-50 mL / min)  4: very severe (> 50 mL / min) |
| Type of hemostasis performed | Manual compression  Clips  Electrocautery  Aquamantys  Ligasure/Harmonic/Thunderbeat/Othe device  Suture  Application of hemostatic agent |
| Hemostatic agents used | **PASIVES**  ***Oxidized cellulose***  Surgicel (Original, Fibrilar, Nuknit, Snow)  Gelitacel  Pahacel  Traumastem  Oxitamp  Kanstat  Reoxcel  Curacel  Equitamp / Equicel  Cellistypt  ***Porcine gelatin***  Gelfoam  Espongostan  Gelita / Gelitaspon  Surgispon  Curaspon  Cutanplast  Hemospon  Surgiflo without thrombin  ***Bovine collagen***  Avitene  Helitene  Hemacoll  Hemotese  Instat  Lyostypt  Novacol  Sangustop  ***Equine collagen***  Collatamp  Genta-Coll  GentaFleece  ***Polysaccharide powders***  PerClot  Arista  4DryField  Haemocer  ***Oxidized cellulose powders***  Surgicel poder  ***Synthetic peptides***  Purastat  **ACTIVES**  ***Fibrin glue***  Tisseel  Veraseal  Vivostat  ***Thrombin and gelatin matrix***  Floseal  Surgiflo with thrombin  ***Advanced patches***  Tachosil  Hemopatch  Veriset |
| Was any hemostatic drug used? | Tranexamic acid  Fresh plasma  Prothrombin complex  Fibrinogen  NovoSeven |

***POSTOPERATIVE COMPLICATIONS***

| Complications | Yes  No |
| --- | --- |
| Clavien-Dindo | I  II  IIIa  IIIb  IVa  IVb  V |
| Comprehensive Complication Index |  |
| Hemorrhage | No  A  B  C |
| Liver failure | Mild  Moderate  Severe |
| Biliary fistula | Yes  No |
| Bilioma | Yes  No |
| Intra-abdominal collection | Yes  No |
| Reintervention | Yes  No |
| Type | Percutaneous drainage  ERCP  Surgical reintervention  Interventional radiology |
| Medical complication | Septic shock  IAM  Cardiac arrest  DVT or pulmonary embolism  Stroke/TIA  Others (specify). |
| Hemoglobin | Preoperative  Lower during the hospital stay |
| Need for IV or PO iron | Yes  No |
| Postoperative transfusion | Yes  No |
| Units of blood transfused |  |
| Units of platelets transfused |  |
| Units of fresh frozen plasma transfused |  |
| Was any hemostatic drug used postoperatively? | Tranexamic acid  Fresh plasma  Prothrombin complex  Fibrinogen  NovoSeven |
| Coagulation status: Lower values during the hospital stay | PT  PTT  INR  TEG |
| Readmission | Yes  No |
| Cause | Specify |

***FOLLOW-UP AT 3 MONTHS***

| Free from disease | Yes  No |
| --- | --- |
| Recurrence date | month/day/year |
| Death | Yes  No |
| Date of death or last visit | month/day/year |
