# Supplementary material for: Evaluation of the validated intraoperative bleeding scale in liver surgery: study protocol for a multicenter prospective study
Source: Front Surg. 2023 Oct 2;10:1223225. doi: 10.3389/fsurg.2023.1223225 (PMC10577188; doi:10.3389/fsurg.2023.1223225)
Supplement: Supplementary File S2 — Schedule of assessments. [file Table2.docx]

**SUPPLEMENTARY FILE S2. Schedule of assessments.**

| **Study periods** | **Entry** | **Intervention** | **Follow-up 30 days** | **Follow-up 90 days** |
| --- | --- | --- | --- | --- |
| Visits | 1 | Daily | 2 | 3 |
| Days | Preoperative | Hospitalization | 30 | 90 |
| Patient information and informed consent | X |  |  |  |
| Patient eligibility confirmation | X |  |  |  |
| Identification data | X |  |  |  |
| Background | X |  |  |  |
| Surgery |  | X |  |  |
| Postoperative |  |  |  |  |
| Follow up |  |  | X | X |
