# Supplementary material for: Evaluation of the validated intraoperative bleeding scale in liver surgery: study protocol for a multicenter prospective study
Source: Front Surg. 2023 Oct 2;10:1223225. doi: 10.3389/fsurg.2023.1223225 (PMC10577188; doi:10.3389/fsurg.2023.1223225)
Supplement: Supplementary File S3 — Participating centers. [file Table3.docx]

**SUPPLEMENTARY FILE S3. Participating centers.**

| **HOSPITAL** | **CITY** |
| --- | --- |
| Hospital Universitario Miguel Servet | Zaragoza |
| Hospital General Universitario Dr. Balmis | Alicante |
| Hospital Universitario Virgen del Rocío | Sevilla |
| Hospital Universitario La Princesa | Madrid |
| Mutua de Terrassa | Tarrasa |
| Hospital Universitario Germán Trias y Pujol | Badalona |
| Hospital Universitario Josep Trueta | Girona |
| Hospital Universitario Infanta Cristina | Badajoz |
| Hospital Clínico Universitario | Valencia |
| Hospital Universitario Gregorio Marañón | Madrid |
