# Supplementary material for: Evaluation of the validated intraoperative bleeding scale in liver surgery: study protocol for a multicenter prospective study
Source: Front Surg. 2023 Oct 2;10:1223225. doi: 10.3389/fsurg.2023.1223225 (PMC10577188; doi:10.3389/fsurg.2023.1223225)
Supplement: Supplementary File S4 — Patient information document and individual informed consent. [file Datasheet1.docx]

**SUPPLEMENTARY FILE S4. Patient information document and individual informed consent.**

**PATIENT INFORMATION DOCUMENT**

**Title:** EVALUATION OF THE VIBe INTRAOPERATIVE BLEEDING SCALE IN LIVER SURGERY. PROSPECTIVE, MULTICENTER STUDY.

**Promoter:**  Aragon Institute for Health Research (IIS Aragon)

**Principal researcher:** Daniel Aparicio López **/** Mario Serradilla Martin / José Manuel Ramia Ángel**.**

**Tfno:** +34 697768059 **e**-mail: [dapariciol@salud.aragon.es](mailto:dapariciol@salud.aragon.es)

**Centro:** General and Digestive Surgery Department. Miguel Servet University Hospital. Zaragoza. Spain

**1. Introduction:**

We are writing to you to request your participation in a research project that we are carrying out at NAME OF THE COLLABORATING HOSPITAL. Your participation is voluntary, but it is important to allow us to obtain the data we need. This project has been approved by the Ethics Committee, but before making a decision you should:

- read this entire document

- understand the information contained in the document

- ask any the questions you consider necessary

- make a thoughtful decision

- sign the informed consent, if you finally want to participate.

If you decide to participate, you will be given a copy of this sheet and the signed consent document. Please keep it in case you need it in the future.

**2. Why are you being asked to participate?**

Your collaboration is requested because you suffer from a disease that affects the liver and requires a surgical intervention. This intervention consists of the resection of the affected liver tissue.

A total of 259 patients with these characteristics will participate in the study.

**3. What is the purpose of this study?**

The study will record and then analyze the number of liver resections performed at 10 moderate-high volume centers (reference centers) in Spain with the intention of determining intraoperative bleeding and validating the VIBe scale for use in liver surgery. Furthermore, we intend to determine the repeatability, reproducibility, and usefulness of the scale in this type of surgery, and the relationship between the extent of bleeding and the hemostatic method used. At present there is no validated scale for assessing intraoperative bleeding in liver surgery, and with this registry we intend to obtain one.

**4. What do I have to do if I decide to participate?**

You do not have to do anything to participate in this registry. You will not have to undergo any diagnostic test other than the normal ones during your diagnostic and therapeutic process. It only authorizes us to quantify and record intraoperative bleeding at two time points of the surgical intervention (the moment of maximum bleeding and at the end of the surgery). Likewise, your medical history will be reviewed to collect the necessary data on the possible complications that you may suffer throughout the process. You do not have to attend more appointments than usual, and you will not be asked to carry out any type of survey.

**5. What risks or discomfort does it entail?**

Participation in this registry will not suppose any type of risk or additional discomfort for the patient.

**6. Will I get any benefit from my participation?**

As this is a research study aimed at generating knowledge, it is not likely that you will obtain any benefit from your participation, although you will be contributing to the advancement of science and the benefit of society.

You will not receive any financial compensation for your participation.

**7. How will my personal data be processed?**

Person responsible for the treatment: Study coordinator. Daniel Aparicio López

Purpose: Your personal data will be processed exclusively for the research work referred to in this document.

Legitimation: The treatment of the data of this study is legitimized by your consent to participate.

All the information collected will be treated in accordance with the provisions of current legislation on the protection of personal data. Personal data will not be included in the study database: neither your name, nor your medical history number nor any information that can identify you. You will be identified by a code that only the research team will be able to associate with your name.

Only the research team will have access to the data in your medical history and no one outside the center will be able to consult your history.

In accordance with the provisions of the data protection legislation (RGPD 2016/679), you can exercise the rights of access, modification, opposition and cancellation of data. You can also limit the processing of data that are incorrect, request a copy or transfer to a third party (portability) the data that you have provided for the study. To exercise your rights, contact the principal investigator of the study. You also have the right to contact the Data Protection Agency if you are not satisfied.

If you decide to withdraw your consent to participate in this study, no new data will be added to the database, but those that have already been collected will be used. Should you want both the data and the samples already collected to be destroyed, you must request this expressly and your request will be attended to.

The coded data may be transmitted to third parties and to other countries but in no case will they contain information that can directly identify you, such as name and surname, initials, address, social security number, etc. In the event that data are transmitted in this way, it will be for the same purposes as those of the study described or for use in scientific publications but always maintaining their confidentiality in accordance with current legislation.

The promoter / researcher will adopt the pertinent measures to guarantee the protection of your privacy and will not allow your data to be crossed with other databases that could allow their identification or use for purposes unrelated to the objectives of this investigation.

The conclusions of the study will be presented in conferences and scientific publications, but they will always be made with grouped data and nothing that can identify it will ever be disclosed.

**8. Who finances the study?**

This study is funded by the Investigator Initiated Research (IIR) grant from Baxter Healthcare Corporation.

**9. Will I be informed of the results of the study?**

You have the right to be informed of the results of this study, both the general results and those derived from your specific data. You also have the right not to know these results if you wish. For this reason, in the informed consent document we will ask you which option you prefer. If you wish to know the results, the researcher will send them to you.

**Can I change my mind?**

Your participation is completely voluntary. You may decide not to participate or withdraw from the study at any time without having to give explanations and without this having an impact on your health care (only for projects in the healthcare field). Simply state your intention to the study's principal investigator.

**What happens if I have any questions during my participation?**

The first page of this document contains the name and contact telephone number of the researcher responsible for the study. You may contact him if you have any questions about your participation.

Thank you very much for your time. If you finally wish to participate, please sign the attached consent document.

**INFORMED CONSENT DOCUMENT**

**Title: EVALUATION OF THE VIBe INTRAOPERATIVE BLEEDING SCALE IN LIVER SURGERY. PROSPECTIVE AND MULTICENTRIC STUDY.**

I, ................................................ .............................. (name and surname of the participant)

have read the information sheet that has been provided to me.

I have been able to ask questions about the study and have received enough information about it.

I have spoken to: .............................................. ............................. (name of researcher)

I understand that my participation is voluntary.

I understand that I may withdraw from the study:

1) whenever I want

2) without having to explain

3) without this affecting my medical care

I freely give my consent to participate in this study and I give my consent for the access and use of my data as stipulated on the information sheet given to me (and for the genetic analysis to be carried out, if applicable).

I wish to be informed about the results of the study: yes / no (check one)

I give my consent for my clinical data to be reviewed by personnel outside the center, for the purposes of the study, and I am aware that this consent is revocable.

I have received a signed copy of this Informed Consent.

Participant signature:

Date:

I have explained the nature and purpose of the study to the patient named.

Investigator's Signature:

Date:
